# Quantitative Evaluation of Improvement of Tear Trough With a Non‐Cross‐Linked Sodium Hyaluronic Compound: A Three‐Dimensional and MRI Analysis

**DOI:** 10.1111/jocd.16718

**Published:** 2025-01-14

**Authors:** Di‐Ya Su, Mu‐Yan Zou, Shi‐Wei Wang, Lei Dong, Jia‐Xu Wu, Xi‐Yue Hu, Jie‐Qing Wang

**Affiliations:** ^1^ Plastic & Aesthetic Surgery Center Dalian University Affiliated Xinhua Hospital Dalian Liaoning China; ^2^ Medical Department Imeik Technology Development Co. Ltd Beijing China

**Keywords:** 3D evaluation, infraorbital hollows, MRI measurement, tear trough, the non‐cross‐linked HA compound

## Abstract

**Background:**

Tear trough deformities are indicative of aging, progressively manifesting as pronounced infraorbital hollows. Although hyaluronic acid (HA) dermal fillers are favored for their safety and efficacy, quantifiable evidence of their effect of action still remains insufficient.

**Aims:**

To investigate the efficacy of non‐cross‐linked HA compound in tear trough enhancement.

**Methods:**

Twenty‐one subjects with moderate to severe infraorbital hollows underwent a single treatment including subcutaneous to supraperiosteal injections of a non‐cross‐linked HA compound. The effectiveness was assessed through Allergan Infraorbital Hollows Scale (AIHS), satisfaction rates, the Global Aesthetic Improvement Scale (GAIS), and quantitative analyses via Canfield VECTRA 3D imaging and MRI measurements during the 6‐month follow‐up.

**Results:**

A total of 0.67 ± 0.31 and 0.77 ± 0.42 mL of the non‐cross‐linked HA compound were injected into the left and right suborbital regions, respectively. Substantial improvements in AIHS were observed in 85.71% and 80.95% of subjects at 6 months posttreatment, as assessed by blinded evaluators and treating investigators, respectively. 3D imaging showed volume increases of 0.87 ± 0.32 (left) and 0.99 ± 0.45 mL (right). MRI analysis provided closely aligned results to the 3D analysis. The GAIS and satisfaction rate were 85.71%, 90.48% for treating investigators and were 90.48% and 95.24% for subjects 6 months posttreatment.

**Conclusions:**

The non‐cross‐linked HA filler demonstrated a safe and efficacious profile for the correction of infraorbital hollows with significant patient satisfaction and sustained outcomes up to 6 months posttreatment. These results support their clinical value in periorbital rejuvenation and reduction of infraorbital hollowing.

## 1 Introduction

The aging process significantly impacts the periorbital region, leading to pronounced changes in facial aesthetics, particularly the development of tear trough deformities, which impart an appearance of fatigue. The skin around periorbital and eyelid area is notably delicate and tends to show signs of aging more rapidly than other facial areas. Correcting these deformities is a primary objective of periorbital rejuvenation.

Histologically, aging in the periorbital is characterized by the presence of skin folds, reduced elasticity, and atrophy of subcutaneous tissues, as well as aging and laxity of supporting structures such as the orbicularis oculi muscle, levator palpebralis, and orbital septum. Additionally, progressive atrophy of muscular and bony structure, along with hyperpigmentation, contribute the severity of tear trough deformities. The reduction in dermal collagen fibers, degeneration of elastic fibers, and loss of subcutaneous fat are the primary factors leading to the formation of wrinkles and volume loss in the skin surrounding the eyes [1, 2]. Thus, targeted improvements in infraorbital hollows are crucial for effective facial rejuvenation, enhancing both integrity and aesthetic appearance.

The correction of infraorbital hollowing can be achieved through various therapeutic approaches, including surgical interventions, dermal fillers injection, and photoelectric techniques. Although surgical treatments are potentially effective, they require prolonged postoperative recovery periods and are associated with higher risk profiles. Photoelectric technology offers a less invasive alternative that can improve fine lines around the eyes, though its effectiveness in correcting moderate to severe infraorbital hollowing is limited. Dermal filler injection with materials such as collagen, hyaluronic acid (HA), hydroxyapatite, and poly‐L‐lactic acid is the preferred method for correcting infraorbital hollows for its preferred efficacy and safety profile.

The infraorbital dermis is characteristically thin, typically < 1 mm in thickness, and contains minimal subcutaneous tissue [3]. This anatomical feature increases the risk of the Tyndall effect and contour irregularities when fillers are administered in this region [4]. Consequently, the choice of an appropriate filler product is critical to optimize outcomes and minimize potential complications. Among available products, the non‐cross‐linked HA compound Hearty (Imeik Technology Development Co. Ltd.) is an optimized choice. It is composed of non‐cross‐linked HA enriched with nutrients such as L‐carnosine, amino acids, and vitamin B_2_. L‐carnosine plays a key role in reducing cellular aging by affecting telomere shortening and mitigating mitochondrial damage. Simultaneously, amino acids and vitamin B_2_ are essential for collagen synthesis, thus providing the necessary building blocks for this crucial process in maintaining dermal integrity, particularly in sensitive areas like the infraorbital region [5].

## 2 Subjects and Methods

### 2.1 Study Design

This study was designed as a single‐center, prospective clinical trial. It received approval from the Ethics Committee (Ethics approval number: 2022‐015‐01) and adhered to the Declaration of Helsinki, along with the specific requirements set by the Ethics Committee.

### 2.2 Population

The inclusion criteria included subjects aged 18–65 with a severity score of 2 (moderate) or 3 (severe) on the Allergan Infraorbital Hollows Scale (AIHS), as assessed by treating investigators. Candidates were required to be willing to undergo HA treatment for tear trough depression and to have voluntarily signed the informed consent form.

Subjects with the following conditions were excluded: lower eyelid retraction or eye pouches in the infraorbital area, allergies to filler materials, noticeable or hypertrophic scars, a predisposition to keloids, current pregnancy or breastfeeding, intention to become pregnant, any fillers or surgical interventions in the treatment area within the last 6 months, and any other conditions deemed unsuitable for participation by the investigators.

Eligible subjects were those who met all the inclusion criteria and did not meet any of the exclusion criteria.

### 2.3 Materials

The non‐cross‐linked HA compound (1.0 mL, Hearty, Imeik Technology Development Co. Ltd.), mainly composed of HA (5.00 mg/mL), L‐carnosine (2.00 mg/mL), glycine (0.10 mg/mL), alanine (0.10 mg/mL), proline (0.20 mg/mL), vitamin B_2_ (0.005 mg/mL), water for injection, and Compound Lidocaine Cream (10g/100 cm^2^, Beijing Ziguang Pharmaceutical Co. Ltd.).

### 2.4 Treatment Procedure

#### 2.4.1 Preoperative Preparation

Before the treatment, all subjects were instructed to remove their makeup and cleanse their faces thoroughly. Photographs of the treatment area were taken and documented. The treating investigators assessed the severity of tear troughs and facial depressions, infraorbital fat protrusion, and skin texture. The areas targeted for injection were marked by treating investigators on the basis of these assessments.

#### 2.4.2 Injection Process

An anesthetic cream was applied to the injection site to minimize discomfort prior to the injection. The injection process began with the needle being accurately positioned vertically down to the surface of the periosteum. It was then carefully maneuvered along the lower edge of the orbit toward the medial canthus. At this juncture, the product was methodically injected, distributing it evenly along the bone surface of the orbital rim within the tear trough area. Subsequently, the injection point was adjusted to the superficial subcutaneous fat layer, where small, evenly spaced injections were administered until the tear trough area appeared smoothly contoured. Following the injection, erythromycin ointment would be applied meticulously to any areas of disrupted skin, and a cold compress was used for 5–10 min to alleviate redness and swelling effectively.

### 2.5 Clinical Assessments

#### 2.5.1 AIHS, GAIS, and Satisfaction Rate

Subjects were followed up at 1, 3, and 6 months posttreatment. The treating investigators assessed the severity of the infraorbital hollows using AIHS at each visit. All subjects received the VECTRA 3D photogrammetry scanning (Canfield Scientific Inc.) before treatment and at each visit. To minimize bias, a blinded evaluator was engaged to score the AIHS scale using the 3D images obtained at each visit [6]. Improvement rates of AIHS were calculated on the basis of the proportion of subjects showing at least a minimal 1‐point improvement on both sides.

Both the treating investigators and the subjects assessed the satisfaction rate and the GAIS improvement rate [7].

The satisfaction were divided into seven categories, ranging from 1 to 7, indicating levels of very dissatisfied, dissatisfied, mildly dissatisfied, barely satisfied, relatively satisfied, satisfied, and very satisfied. The satisfaction rate is defined as the proportion of treating investigators (subjects) who rated mildly dissatisfied or above.

The GAIS was employed to objectively assess the overall clinical outcomes. Both the treating investigators and the subjects rated the treated area on the scale where “worse than before” received a score of −1, “clinically unchanged” received a 0, “slightly improved” received a 1, “moderately improved” received a 2, “markedly improved” received a 3, and “near totally improved” received a 4. The GAIS improvement rate is defined as the proportion of treating investigators (subjects) who rated 3 or above.

### 2.6 VECTRA 3D Imaging and MRI

The blinded evaluator assessed volumetric changes using Face Sculptor software (Canfield Scientific Inc.) on the 3D images from each visit. Facial MRI scans were conducted on voluntary subjects who had provided the informed consent at 1 week, 1 month, 3, and 6 months posttreatment. MRI analysis would be conducted to assess the condition of the infraorbital filler. Computer‐assisted tools would be used to perform three‐dimensional reconstruction of the 3D MPRAGE images, and MATLAB software would be utilized to delineate the tissue boundaries. By analyzing the temporal changes in HA signal intensity on fat‐saturated T2‐weighted images, the distribution, location, and volume of the filler were evaluated. Quantitative evaluation of volume changes in the infraorbital region was performed by combining 3D analysis with MRI results.

### 2.7 Statistical Analysis

Data analysis was performed using SPSS version 27.0 (IBM Corporation, Armonk, New York, USA). Continuous data, including baseline information, were presented as mean ± standard deviation (SD), assuming a normal distribution. If the distribution deviated from normality, median and interquartile ranges would be used. To determine whether there were significant differences in volumetric changes at 1, 3, and 6 months posttreatment, Friedman's two‐way analysis of variance by ranks was applied, followed by Conover's post hoc test for pairwise comparisons among the time points.

## 3 Results

### 3.1 Participants Disposition and Characteristics

A total of 21 eligible subjects (19 females and 2 males) with infraorbital hollows were enrolled between March 2022 and December 2022. Each subject underwent a single treatment of the non‐cross‐linked HA compound and was followed up for 6 months to assess safety and efficacy.

All 21 enrolled subjects completed the follow‐up visits at 3 and 6 months posttreatment, whereas only 13 completed the follow‐up visits at 1 month because of disruptions caused by a COVID‐19 outbreak between December 2002 and January 2003.

Baseline demographic data are detailed in Table 1. The median age was 41.0 ± 10.9 years. Among these subjects, 14 (66.67%) were categorized as AIHS grade 2 and 7 (33.33%) as AIHS grade 3.

**TABLE 1:** Demographic and Clinical Characteristics.

Characteristic	Subjects, no.	
Age, mean (range)	41.0 ± 10.9	
Gender, no. (%)	Male 2 (9.5%); Female 19 (90.5%)	
Infraorbital hollows grade	Left	Right
2 (Moderate)	14 (66.67%)	14 (66.67%)
3 (Severe)	7 (33.33%)	7 (33.33%)
Average injection volume	0.67 ± 0.32 mL	0.77 ± 0.41 mL

All subjects received a single injection of the non‐cross‐linked HA compound. The average volumes injected were 0.67 ± 0.31 mL (ranging from 0.30 to 1.60 mL) and 0.77 ± 0.41 mL (ranging from 0.20 to 1.70 mL) into the left and right suborbital regions, respectively.

### 3.2 AIHS, GAIS, and Satisfaction Rate

The AIHS improvement rate of treating investigators indicated that 84.62% of the subjects had AIHS improvement at 1 month after injection, and 80.95% of them maintained the improvement at 3 months and 6 months posttreatment.

Blinded evaluators demonstrated an 84.62% improvement rate at the 1 month follow‐up, and an 85.71% improvement rate at both the 3‐ and 6‐month follow‐ups (Table 2).

**TABLE 2:** AIHS improvement rate by treating investigators and blinded evaluators.

AIHS improvement rate	Month 1 (*N* = 13)	Month 3 (*N* = 21)	Month 6 (*N* = 21)
Treating investigator	84.62% (11/13)	80.95% (17/21)	80.95% (17/21)
Blinded evaluator	84.62% (11/13)	85.71% (18/21)	85.71% (18/21)

**FIGURE 1:**
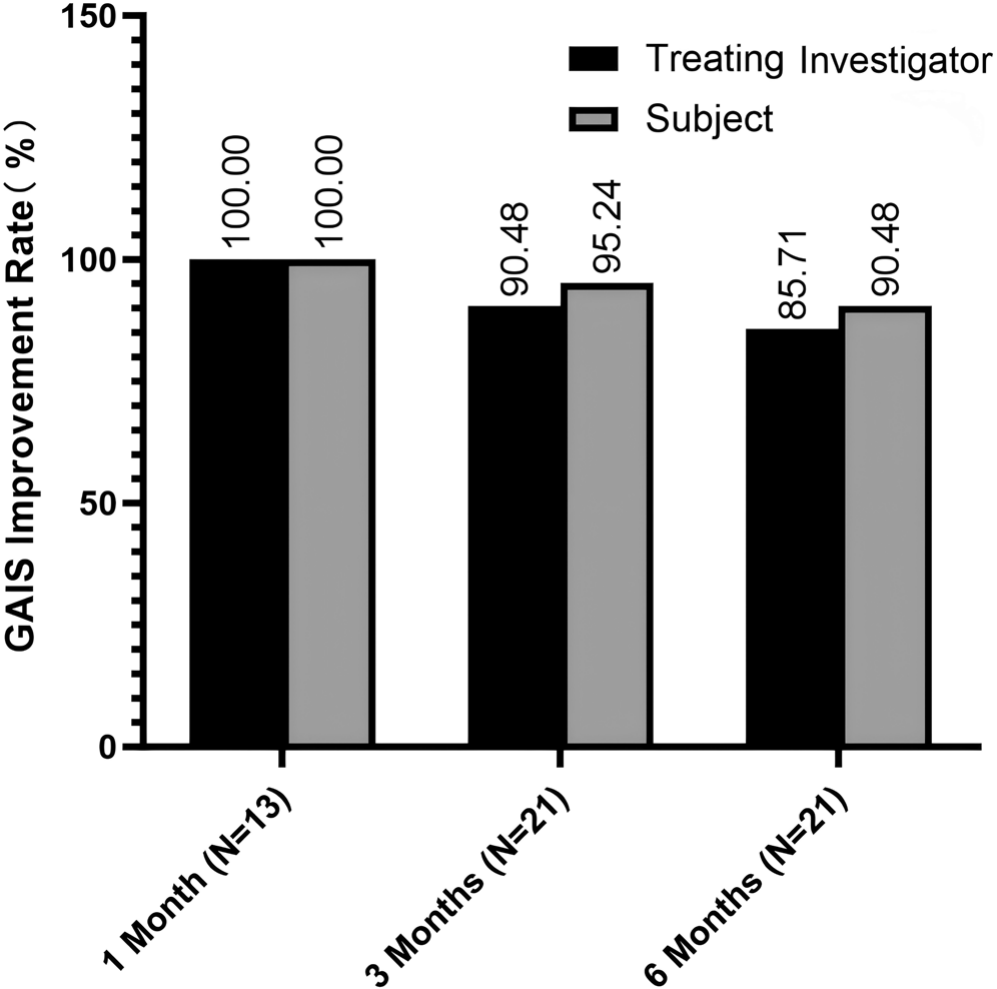
Bar diagram showing the Global Aesthetic Improvement Scale (GAIS) improvement rates in % for treating investigators (black) and subject (gray).

**FIGURE 2:**
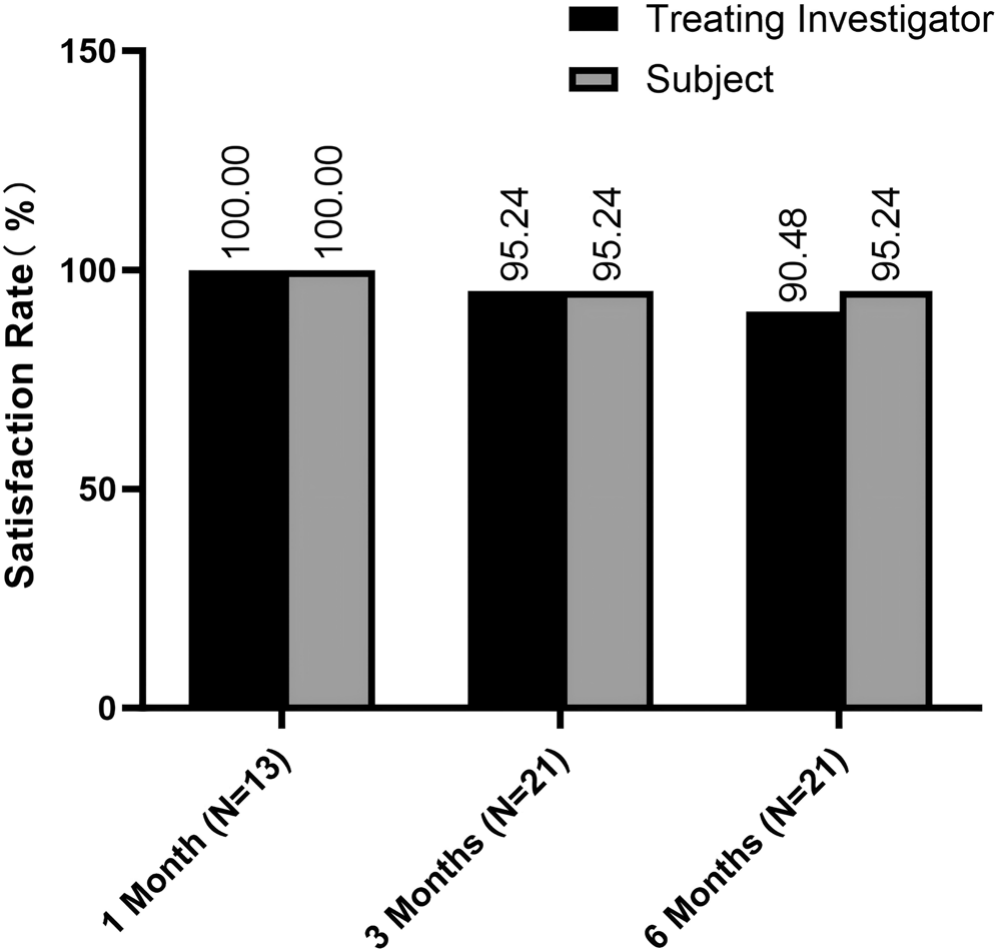
Bar diagram showing the satisfaction rates in % for treating investigators (black) and subject (gray).

According to GAIS scores reported by treating investigators, all subjects (100%) showed improvement at the 1‐month follow‐up, with improvement maintained by 90.48% at 3 months and 85.71% at 6 months after injection. Similarly, self‐reported GAIS scores showed improvement in 100% of subjects at 1 month, 95.24% at 3 months, and 90.48% at 6 months posttreatment (Figures 1 and 2).

The satisfaction rate was 100.00% at the 4‐week follow‐up, 95.24% at both the 12‐week follow‐up and 24‐week follow‐up is indicated as 95.24%. The alignment between the satisfaction levels reported by subjects and those assessed by evaluators underscores a significant agreement on the positive impact of the treatment, sustaining over the 6‐month follow‐up period.

### 3.3 Canfield VECTRA 3D Imaging Measures Suborbital Volumetric Changes

**FIGURE 3:**
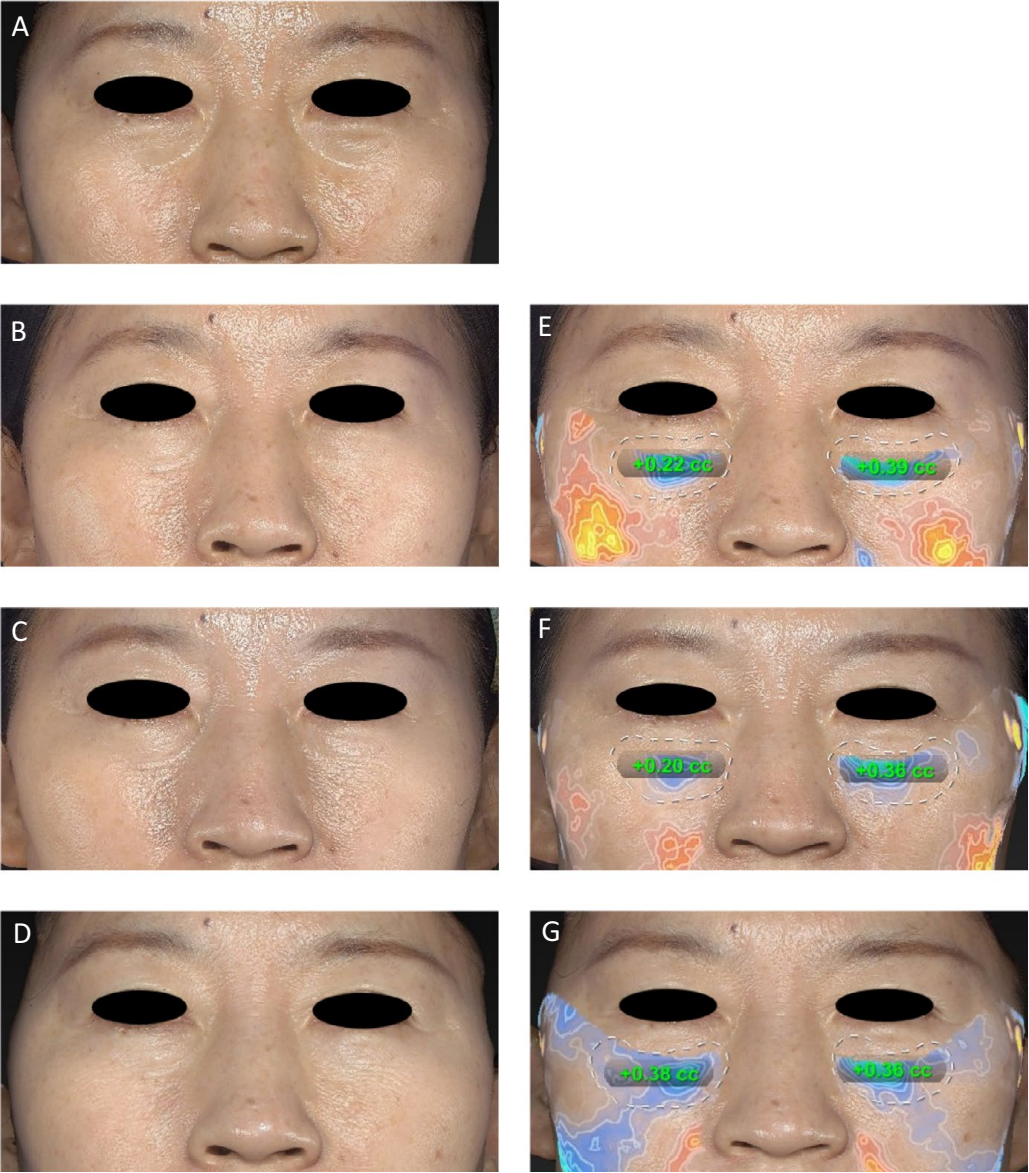
Three‐dimensional photographs (Canfield VECTRA 3D imaging) evaluating the volumetric changes in the right and left sides of suborbital of a 64‐year‐old woman who underwent treatment with Hearty. (A) At baseline with AIHS score = 3 (severe, both sides), (B) at Month 1 with AIHS score = 1 (minimal, left side); 2 (moderate, right side), and (C) at Month 3 with AIHS score = 1 (minimal, left side); 2 (moderate, right side), and (D) at Month 6 with AIHS score = 1 (minimal, both sides), following initial treatment volume of 0.6 mL (left side) and 0.4 mL (right side) total. Panels (E), (F), and (G) represent volumetric difference maps generated by the Vectra 3D analysis software, corresponding to the same time points shown in B, C, and D, respectively, thereby illustrating the changes in suborbital volume.

**FIGURE 4:**
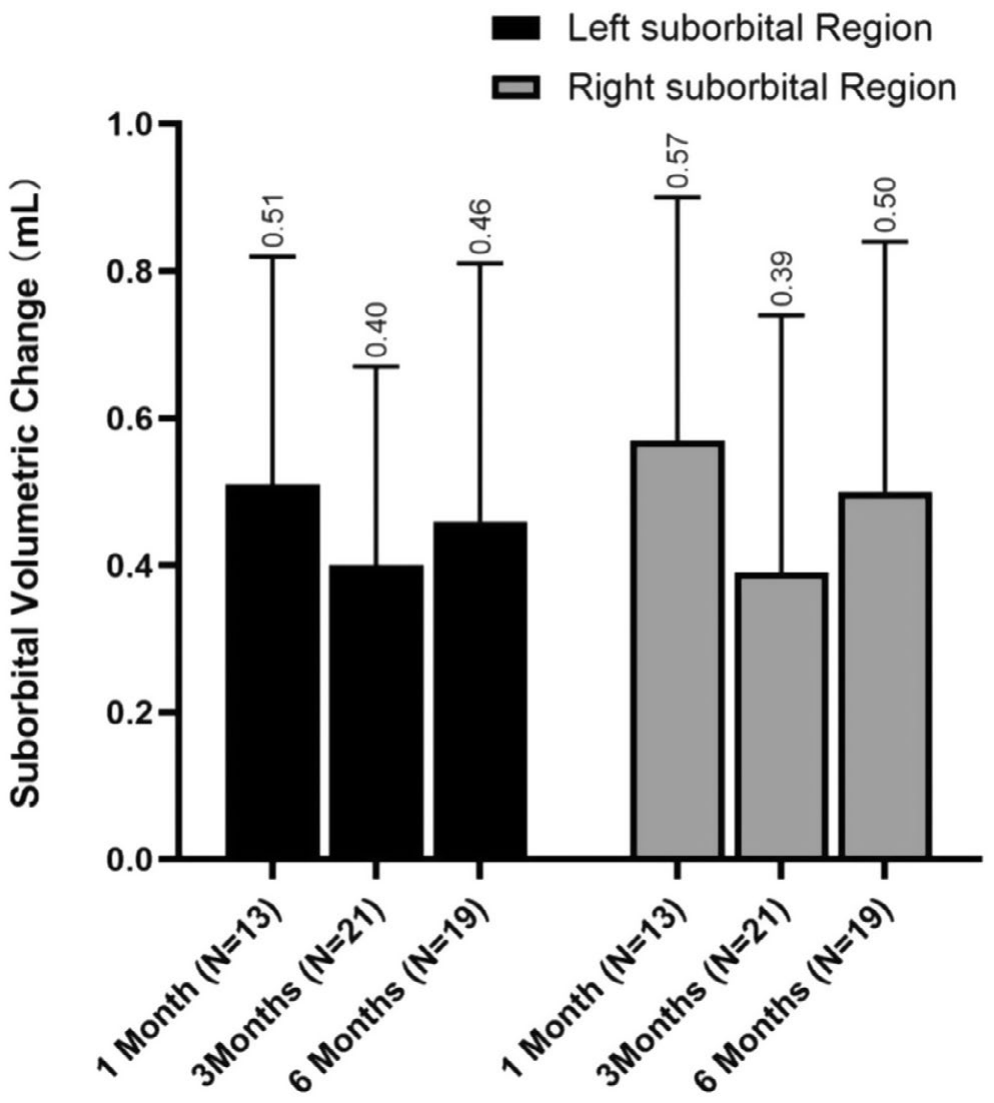
Mean change from baseline of suborbital volumetric change.

At 1 month after treatment, an average volume increase of 0.51 ± 0.31 mL in the left suborbital region and 0.57 ± 0.33 mL in the right suborbital region were observed. By the 3‐month follow‐up, the mean volume change was slightly reduced to 0.40 ± 0.27 mL in the left suborbital region and 0.39 ± 0.35 mL in the right suborbital region. And by 6‐month follow‐up, the average volume adjustment in the left suborbital region was noted as 0.46 ± 0.35 mL, compared with 0.50 ± 0.34 mL for the right suborbital region (Figure 3). The Friedman test results showed nonsignificant difference on left (*p* = 0.198). For the right side, volume change at 6 months posttreatment was significantly greater than that at 3 months after injection (*p* = 0.021), whereas no significant differences were observed between 1 and 6 months posttreatment. Additionally, there were no significant differences between the left and right at 1, 3 and 6 months after injection (Figure 4).

### 3.4 MRI Analysis

**FIGURE 5:**
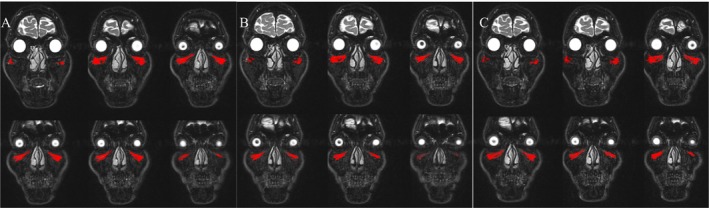
The coronal T2‐weighted fat‐saturated 3‐T MRI scan of a male subject, age 27 years, achieved a 0‐point improvement on the AIHS following treatment with Hearty. Red highlights indicates the signal of volume enhancement in the infraorbital region. (A) 1 weeks after injection, (B) 1 months after injection, and (C) 3 months after injection.

The efficacy and distribution of HA were evaluated using MRI. A total of four subjects provided the informed consents to receive MRI scans. One of four subjects failed to receive the MRI scans because of the COVID‐19 and was excluded. On fat‐saturated T2‐weighted scans, the HA and its associated bound water exhibited optimal visualization on fat‐saturated T2‐weighted scans, characterized by high signal intensity areas with clearly delineated serpiginous margins. The MRI images showed that the HA compound were uniformly distributed across the bilateral infraorbital region (Figure 5).

**FIGURE 6:**
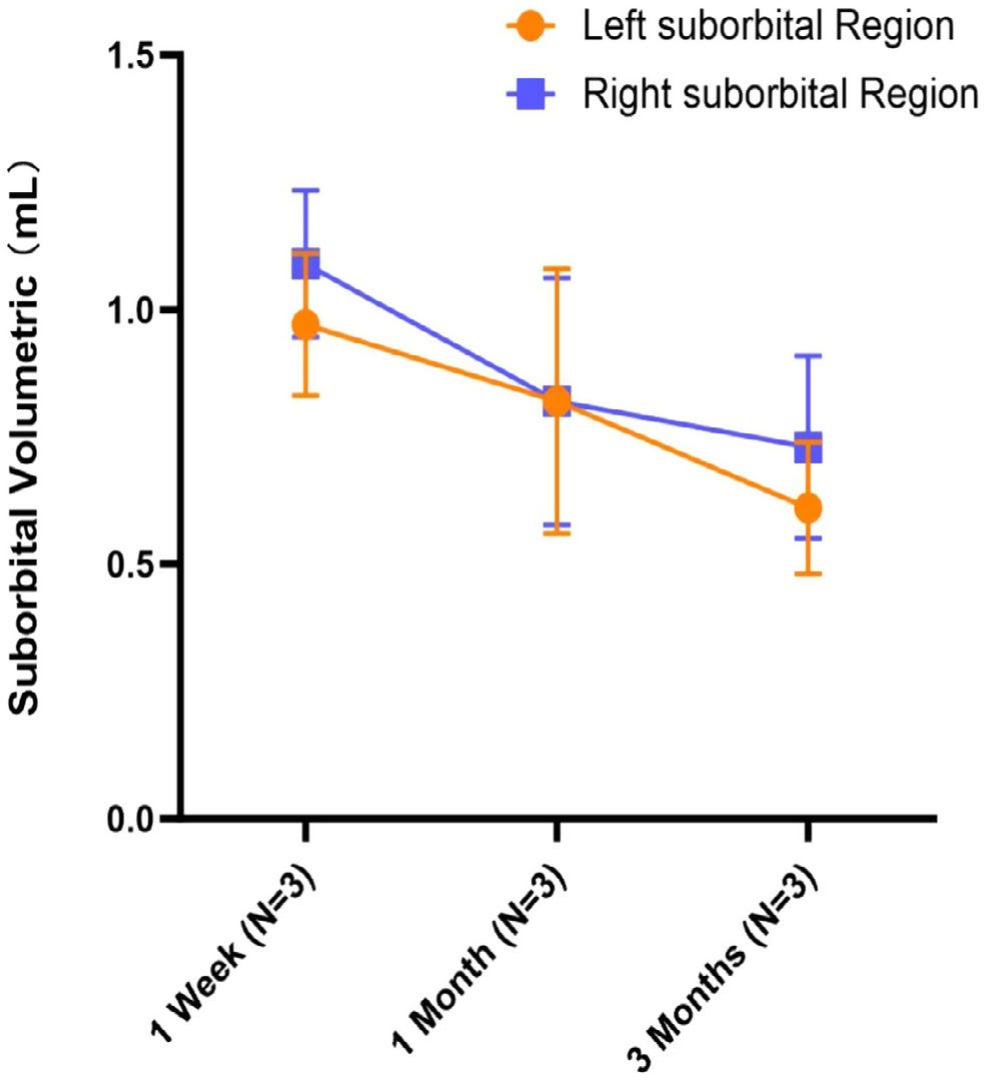
Mean volumes of left (yellow) and right (blue) suborbital region measured by MRI 1 week, 1, and 3 months after injection.

The mean (±SD) volumes for the left side were 0.97 ± 0.14 mL at 1 week, 0.82 ± 0.26 mL at 1 month, and 0.61 ± 0.13 mL at 3 month. The mean (±SD) volumes for the right side were 1.09 ± 0.25 mL at 1 week, 0.82 ± 0.42 mL at 1 month, and 0.73 ± 0.31 mL at 3 month (Figure 6). MRI analysis indicated that the application of non‐cross‐linked HA maintained substantive volume enhancement in the infraorbital region for at least 3 months, with notable volume retention. The quantitative MRI findings were consistent with the volumetric trends observed in the 3D reconstructions.

### 3.5 Safety

No serious adverse event was reported. Three subjects (14.29%) experienced transient mild swelling at injection site, which resolved within a week without requiring additional intervention. Furthermore, no cases of delayed granulomas or the Tyndall effect were observed throughout the follow‐up period.

## 4 Discussion

As individuals age, the tear troughs at the inferior orbital margin extend along or toward the lateral side, enhancing the signs of aging and imparting a tired appearance to the infraorbital area. The formation of infraorbital hollows is closely associated with the aging process of the skin, fat, ligaments, orbicularis oculi muscle, orbital septum, and bone structure. These suborbital volume defects can be effectively remediated through minimally invasive injectable fillers such as autologous fat transplantation, collagen supplementation, HA injections, application of platelet‐rich plasma, and poly‐L‐lactic acid treatments. These methods have demonstrated efficacy in restoring volume and reducing the visible signs of aging in the periorbital region, as supported by numerous clinical studies [8].

Among various injectable materials available, the HA filler is particularly notable for its broad acceptance and multiple benefits because of minimal invasiveness, reduced treatment trauma, immediate visible results, and the possibility for straightforward adjustments postinjection [4, 9].

Given the intricate anatomical and vascular complexities of the ocular region, the selection of appropriate injectable products and precise determination of their injection depth, while cognizant of their rheological and physicochemical attributes, are crucial [10].

The infraorbital region is characterized by the delicate skin and limited fat tissues. Although most HA fillers currently in use are cross‐linked, the thin dermis and minimal subcutaneous fat in the infraorbital area makes the application of cross‐linked HA more susceptible to forming nodules beneath the skin [11, 12]. Injection of cross‐linked fillers may contribute to undesirable outcomes, including hematoma, edema, uneven texture, and color discrepancies because of the Tyndall phenomenon [13, 14]. Additionally, non‐cross‐linked HA reduces the risk of vascular obstruction, and also minimizes the potential for facial overfill syndrome. The non‐cross‐linked HA compound (Hearty, Imeik Technology Development Co. Ltd.) is primarily composed of non‐cross‐linked HA. Compared with traditional cross‐linked forms, non‐cross‐linked HA exhibits offer superior spreadability and reduces the risk of complications such as vascular occlusion and formation of lumps often associated with infraorbital filling. In addition, the non‐cross‐linked HA compound also supplied L‐carnosine, glycine, proline, hydroxyproline and Vitamin B_2_, playing a part in antioxidant, promoting new collagen production and maintaining the integrity of skin tissues [13, 14, 15, 16]. Previous studies had approved that the complex formula of the non‐cross‐linked HA compound provides the immediate adjustments of injection site whereas collagen synthesis and tissue repair by stimulating the proliferation and motility of fibroblasts and improvement of appearance and texture of the skin [15, 16].

In recent years, the field of medical cosmetology has seen significant advances with the introduction and application of new detection technologies such as skin imaging, 3D scanning, MRI, CT, and ultrasound. These technologies have markedly improved the ability to quantitatively analyze the effects of postimplantation fillers, both in clinical practice and research settings [17].

Canfield VECTRA 3D imaging and Sculptor software enable the treating investigators quantitatively assesses vector motion, volume variations, and additional parameters in targeted body areas [18, 19]. MRI assists in evaluating the localization, dispersion, volume fluctuations, signal variances, degradation rates, and epidermal thickness consequent to filler injections and aids in tracking possible adverse outcomes or complications postinjection, like nodules or granulomas. When integrated with VECTRA 3D technology, MRI provides a more objective and comprehensive evaluation of the effectiveness and safety of filler treatments.

In this study, we employed a comprehensive assessment that combined VECTRA 3D imaging analysis, MRI analysis, and subjective evaluation scales to evaluate both the volume enhancement and durability of infraorbital soft tissue augmentation in the subjects.

The VECTRA 3D analysis revealed that the subjects experienced the aesthetic enhancement 6 months after injection. The initial (1 month after injection) significant volumetric augmentation in infraorbital area was largely due to the hydrating effects of HA. However, because of the degradation property of the non‐cross‐linked HA, a volume decrease was observed at 3 months posttreatment. During this period, there was a notable surge in cellular activity, evident by the stimulated growth of fibroblasts and the formation of new blood vessels. Molecular evidence also shown that the skin rejuvenation and anti‐wrinkle effects of HA are attributed to its ability to stimulate collagen synthesis by activating fibroblasts in the dermis. The increased collagen production results in smoother skin, reduced wrinkles, and enhanced skin elasticity and durability [20].

Thus, even though the HA was naturally hydrolyzed and absorbed, the volume loss was effectively counteracted by the production of connective tissue, fat cells, and new blood vessels from natural physiological processes, illustrating the remarkable tissue regeneration capabilities at 6 months after injection. Although, the MRI analysis results provided closely aligned results to the VECTRA 3D analysis, confirming the consistency and similarity of the volumetric findings.

The non‐cross‐linked HA compound has favorable safety profile, with only about 14.29% of subjects experiencing transient mild swelling, which resolved within 1 week. Notably, the challenging Tyndall phenomenon, often associated with periorbital injections, was not observed in this study.

In conclusion, the effectiveness and safety of the non‐cross‐linked HA compound for treating infraorbital hollowing were substantiated through comprehensive assessments through VECTRA 3D, MRI analysis, and subjective evaluations. The treatment yielded positive, enduring, and satisfactory outcomes, with the benefits persisting for at least 6 months. Importantly, it only induced minor and transient injection‐related reactions, with no serious adverse effect reported during the follow‐up period, thereby affirming its suitability for periorbital rejuvenation.

Although providing valuable insights, this study also had several limitations. Long‐term effects of the HA compound treatment may not be provided by the short follow‐up period (6 months). Additionally, the small sample size, coupled with an unequal gender distribution, might not have yielded results that are generalizable across a broader population. Subjects with severe infraorbital hollowing could potentially require multiple treatments to achieve optimal outcomes. The nonrandomized design and the absence of a control group further constrained the conclusiveness of our findings.

## Author Contributions

Di‐Ya Su and Xi‐Yue Hu performed the research. Mu‐Yan Zou and Di‐Ya Su designed the research study. Jie‐Qing Wang, Shi‐Wei Wang, and Lei Dong contributed essential reagents or tools. Di‐Ya Su and Jia‐Xu Wu analyzed the data. Di‐Ya Su and Jia‐Xu Wu wrote the paper. Each author have participated sufficiently in the work to take public responsibility for appropriate portions of the content and agreed to be accountable for all aspects of the work. All authors have read and approved the final manuscript.

## Ethics Statement

The study was approved by the Dalian University Affiliated Xinhua Hospital Ethics Committee and complied with the ethical requirements of the Declaration of Helsinki.

## Conflicts of Interest

The authors declare no conflicts of interest.

## Data Availability

The data that support the findings of this study are available from the corresponding author upon reasonable request.
